# Administration of quercetin improves mitochondria quality control and protects the neurons in 6-OHDA-lesioned Parkinson's disease models

**DOI:** 10.18632/aging.202868

**Published:** 2021-04-20

**Authors:** Wen-Wen Wang, Ruiyu Han, Hai-Jun He, Jia Li, Si-Yan Chen, Yingying Gu, Chenglong Xie

**Affiliations:** 1 The Center of Traditional Chinese Medicine, The Second Affiliated Hospital and Yuying Children's Hospital of Wenzhou Medical University, Wenzhou 325027, China; 2 Department of Neurology, The First Affiliated Hospital of Wenzhou Medical University, Wenzhou 325000, China; 3 Department of Psychiatry, The First Affiliated Hospital of Wenzhou Medical University, Wenzhou 325000, China; 4 NHC Key Laboratory of Family Planning and Healthy, Hebei Key Laboratory of Reproductive Medicine, Hebei Research Institute for Family Planning Science and Technology, Shijiazhuang 050071, Hebei, China

**Keywords:** quercetin, Parkinson's disease, mitophagy, mitochondria quality control

## Abstract

Mounting evidence suggests that mitochondrial dysfunction and impaired mitophagy lead to Parkinson’s disease (PD). Quercetin, one of the most abundant polyphenolic flavonoids, displays many health-promoting biological effects in many diseases. We explored the neuroprotective effect of quercetin *in vivo* in the 6-hydroxydopamine (6-OHDA)-lesioned rat model of PD and *in vitro* in 6-OHDA-treated PC12 cells. *In vitro*, we found that quercetin (20 μM) treatment improved mitochondrial quality control, reduced oxidative stress, increased the levels of the mitophagy markers PINK1 and Parkin and decreased α-synuclein protein expression in 6-OHDA-treated PC12 cells. Moreover, our *in vivo* findings demonstrated that administration of quercetin also relieved 6-OHDA-induced progressive PD-like motor behaviors, mitigated neuronal death and reduced mitochondrial damage and α-synuclein accumulation in PD rats. Furthermore, the neuroprotective effect of quercetin was suppressed by knockdown of either *Pink1* or *Parkin*.

## INTRODUCTION

Parkinson's disease (PD) is characterized by progressive and selective loss of dopaminergic neurons in the substantia nigra (SN) [1, 2]. In terms of the pathological mechanism, α-synuclein (a-syn) is a major ingredient of intracellular inclusion Lewy bodies, which are a neuropathological hallmark of PD. The presence of a-syn aggregates in PD patient brains indicates that the proteostasis of α-syn is impaired and contributes to the role of aging in PD pathophysiology [3]. Currently, levodopa (L-dopa) is still a mainstay therapy utilized in the treatment of PD subjects to try to compensate for the lack of dopamine and dopaminergic functions. Nevertheless, the shortcomings of this pharmacotherapy include medication-related motor complications containing motor fluctuations, dyskinesias etc., and diminished effectiveness over time. In the past decade, researchers have made pioneering discoveries in several areas, including progressive dopamine neuron death in PD, α-syn homeostasis, oxidative damage, impaired autophagy or mitophagy and the loss of calcium homeostasis [4]. Among them, impaired mitophagy may be a promising novel target for pharmacological manipulation [5].

Mitophagy is the selective clear away defective or redundant mitochondria through the autophagy-lysosome pathway and is the best-studied type of selective autophagy [6]. In the past decade, the underlying molecular mechanisms of mitophagy have been comprehensively explored, particularly the PINK1–Parkin-mediated mitophagy pathway [7]. Notably, the accumulation of damaged mitochondria results in the death of dopaminergic neurons, and mitophagy plays a vital role in removing these mitochondria from cells [8]. Targeting this pathway is a therapeutic strategy for a couple of neurodegenerative diseases, most notably PD [9]. In addition, mutations in the genes that encode PINK1 and Parkin cause early-onset PD [10]. Dysfunction of the PINK1 and Parkin proteins has also been reported in sporadic PD [11]. Enhancing the ability to degrade defective or superfluous mitochondria may prevent the degeneration of dopaminergic neurons and delay disease progression. Indeed, upregulation of PINK1 competence using the neosubstrate KTP was shown to increase the survival of dopaminergic neurons challenged with oxidative stress [12].

Quercetin is an important flavonoid and polyphenol that is ubiquitously present in the diet and is found in many fruits and vegetables, including capers, figs, cranberries, Patel et al. [13]. To now, quercetin has been reported to have lot of pharmacological functions, including anti-inflammatory, antidiabetic, neuroprotective, antiobesity and anticancer activities. The main molecular mechanism responsible for its neuroprotection is its capacity to quench reactive oxygen species (ROS) and hence reduce oxidative damage, which is responsible for the development of diverse diseases [14, 15]. Kwon et al. reported that quercetin-3-O-galactoside suppressed neuronal death caused by 6-OHDA-induced oxidative stress via the induction of nuclear erythroid 2-related factor 2 (Nrf2)-dependent heme oxygenase-1 (HO-1) activation [16]. Treatment with quercetin defended against oxidative stress in the striatum and alleviated dopaminergic neuronal loss in a PD rat model [17]. Moreover, recent research has shown that quercetin may exert beneficial effects independent of its free radical-scavenging properties. Wang et al. demonstrated that quercetin induced protective autophagy in gastric cancer cells through Akt-mTOR- and hypoxia-induced factor 1α (HIF-1a)-mediated signaling [18]. In ovarian cells, activating the p-STAT3/Bcl-2 axis via quercetin administration leads to autophagy induction and inhibits disease progression [19]. Recently, some attention has been focused on the effect of quercetin on mitochondrial biogenesis, oxidative respiration, mitochondrial membrane potential (MMP), mitochondrial quality control and adenosine triphosphate (ATP) anabolism [20]. However, the detailed mechanism of the effect of quercetin in mitophagy or mitochondrial quality control is still elusive. Hence, the present study aimed to investigate whether quercetin protects against mitochondrial damage in response to toxicity induced by the 6-OHDA stressor.

## MATERIALS AND METHODS

### Cell culture and treatment

PC12 cells utilized in this study to explore the protective role of quercetin against the 6-OHDA (Sigma-Aldrich) *in vitro*. The cells were cultured in Dulbecco's modified Eagle medium (DMEM) with 10% fetal bovine serum (FBS, Gibco), supplemented with 1% antibiotic (Gibco). PC12 cells were divided into the following groups: Control group, 6-OHDA-lesioned group (25, 50, 100, 150 and 200 μM, respectively), quercetin (Sigma-Aldrich) group (1, 10, 20, 50 and 100 μM, respectively). PC12 cells were treated with 6-OHDA for 24 hours to induce the PD model *in vitro*. Quercetin (purity > 98%) was pretreatment for 4 h and then co-operated with the 6-OHDA together for 24 h again. Quercetin was dissolved in DMSO.

### Determination of cell viability and small interfering RNAs (siRNA) experiments

CCK-8 kit (catalog no.C0038; Beyotime company, China) was adopted for cytotoxicity assay. Briefly, cell suspension (1.0 × 10^6^/ml) in a 96-well plate was pre-incubated for 24–48 h. Quercetin was administered 4 h before the application of 6-OHDA, and then together 24 h. Then, 10 μl CCK-8 solution was added to each well of the plate and incubated for 1 h at 37°C. Then optical density was measured by spectrophotometry at 450 nm. To silence PINK1 and Parkin expression, transfection of siRNAs was conducted using the Lipofectamine 2000 reagent. The PINK1 and Parkin-specific package of four siRNAs was purchased from Origene (catalog no.SR324912 and SR303355, respectively), and scrambled (negative control) siRNA (catalog no.SR30004) was also obtained from Origene.

### Mitochondrial parameters assay

PC12 cells were plated in 96-well plates at a density of 1 × 10^4^ cells/well. After 24 h, cells were treated with indicated dose quercetin for 24 h. Cells were then washed with serum-free medium and incubated with MitoTracker Green FM (M7514, ThermoFisher) for the quantification of mitochondrial content, MitoSOX (M36008, ThermoFisher) to test the ROS levels and TMRM (T668, ThermoFisher) to assess the MMP, all of them incubated for 30 min based on their manual protocols. The fluorescence was read on a fluorescence microplate reader (excitation 485 nm, emission 520 nm).

### Detection of mitophagy *in vitro* and *in vivo*

*In vitro,* imaging of mt-Keima HeLa cells to test the mitophagy induction, was conducted as reported by Nuo and colleagues [21], utilizing different settings for Green fluorescent protein (GFP) and red fluorescent protein (RFP). Keima is a ratiometric pH-sensitive fluorescent protein which exhibits green fluorescence in neutral conditions and pH-insensitive fluorescent protein which shows red fluorescence in acidic conditions. Mitophagy was calculated as RFP/(GFP + RFP). When mitochondria are transmitted to the acidic lysosomal environment, color shift enables reflecting the level of mitophagy and also assessment of mitophagic flux [22]. For the detailed way how Keima was introduced into the HeLa cells according to previously described paper [23]. *In vivo*, regarding mitophagy in *C. elegans* was measured using the neuronal mt-Rosella [24]: transgenic nematodes expressing a pan-neuronal mt-Rosella biosensor that combines a pH-sensitive GFP variant and fused to the pH-insensitive DsRed. Mitophagy value was reflected as GFP/DsRed.

### Animals and administration

All the procedures involving animal studies had been approved by the Animal Experimental Ethical Committee of Wenzhou Medical University. For the first experiment part, thirty-two adult male Sprague-Dawley (SD; 3–4 months old, 250–300g) rats were randomly divided into four groups (*n* = 8/group), receiving a daily saline vehicle or quercetin (10 mg/kg/d and 30 mg/kg/d) administration over 14 days by oral gavage. For the second experiment part, twenty-four male SD rats were randomly allocated into four groups (*n* = 6 per group) as follows: 6-OHDA-lesiond PD group (vehicle); PD group plus quercetin (30 mg/kg/d); PD group plus quercetin (30 mg/kg/d) plus AAV-*pink1*-shRNA; PD group plus quercetin (30 mg/kg/d) plus AAV-shRNA. After the AAV injection, we should wait three weeks to induce the transfection effects. Based on our previous paper [25], viruses containing silence *pink1* vector was infused stereotactically into the unilaterally striatum of lesioned side (0.1 ul/min for 10 min). Finally, the titer used in the study had been diluted to 1.37E + 12 v.g./ml. Moreover, the coordinates relative to Bregma as follows: 1) anterior-posterior, +0.9 mm, medial-lateral, -4.5 mm, dorsal-ventral, -5.0 mm relative to Bregma; 2) anterior-posterior, +0.5 mm, medial-lateral, -2.5 mm, dorsal-ventral, -4.2 mm according to the rat brain atlas.

### Induction of 6-OHDA-lesiond PD models and behaviors test

SD Rats were anesthetized with 1% pentobarbital sodium (40 mg/kg, i.p.) prior to the surgical process, and then installed on a stereotaxic apparatus, which was consistent with our previous publication papers [26, 27]. Two weeks after surgery, rats were screened out by the rotations after the use of apomorphine (Wako Co. Ltd, 0.5 mg/kg, i.p.) to select the successful PD rats. To assess motor behavior, rotarod test, forelimb function test and Apomorphine induced rotation were used every day for one week to measure the locomotor activity of rats, as described previously in our publications [27]. Before treatments, mice were trained for three consecutive days. Each rat was tested 3 times with an interval of 15 minutes.

### Determination of MDA, ROS and SOD levels

Unilateral substantia nigra were removed from each brain. MDA (catalog no. S0131, Beyotime, China), ROS (catalog no. S0033, Beyotime, China) and SOD (catalog no. S0103, Beyotime, China) levels were determined using corresponding commercial kits according to the instructions. Protein concentration of the sample was determined using a BCA protein assay kit (catalog no. P0012; Beyotime, China).

### Western blot

The second day after the behavior tests, immunoblot analysis was performed as previously described [28]. Briefly, proteins were extracted from the ipsilateral midbrain using Tissue Protein Extraction Reagents. Equal amounts of proteins were loaded and resolved through SDS-polyacrylamide gel electrophoresis and transferred onto PVDF membranes. The membranes were then incubated with antibodies as follows: anti-Tyrosine Hydroxylase (TH) antibody (catalog no. ab5968; Millipore), anti-a-Synuclein (catalog no.04-1050; Millipore), Pink1 antibody (catalog no. ab75487, Abcam); Parkin antibody (catalog no.NB100-91921; Novus); β-actin antibody (catalog no. A5441; Sigma); phospho-TBK1 antibody (catalog no.5483s; CST); TBK1 antibody (catalog no.3504s; CST); phospho-ULK1 antibody (catalog no.5869s; CST); ULk1 antibody (catalog no.6439s; CST), and then followed by horseradish peroxidase-labeled IgG. The membranes were developed using enhanced chemiluminescence detection reagents.

### Statistical analysis

Prism 8.0 was used for the statistical analysis. All *in vitro* data were decided from at least two biologically repeat experiments. The data are expressed as mean ± sem. Statistical analysis was carried out using one-way analysis of variance (ANOVA), followed by Dunnett's test to assess the statistical significance between different groups, or two-way ANOVA test followed by Bonferroni’s post hoc test. A level of *P* < 0.05 was considered statistically significant.

## RESULTS

### Quercetin induces the mitophagy ability *in vitro* and *in vivo*

**Figure 1:**
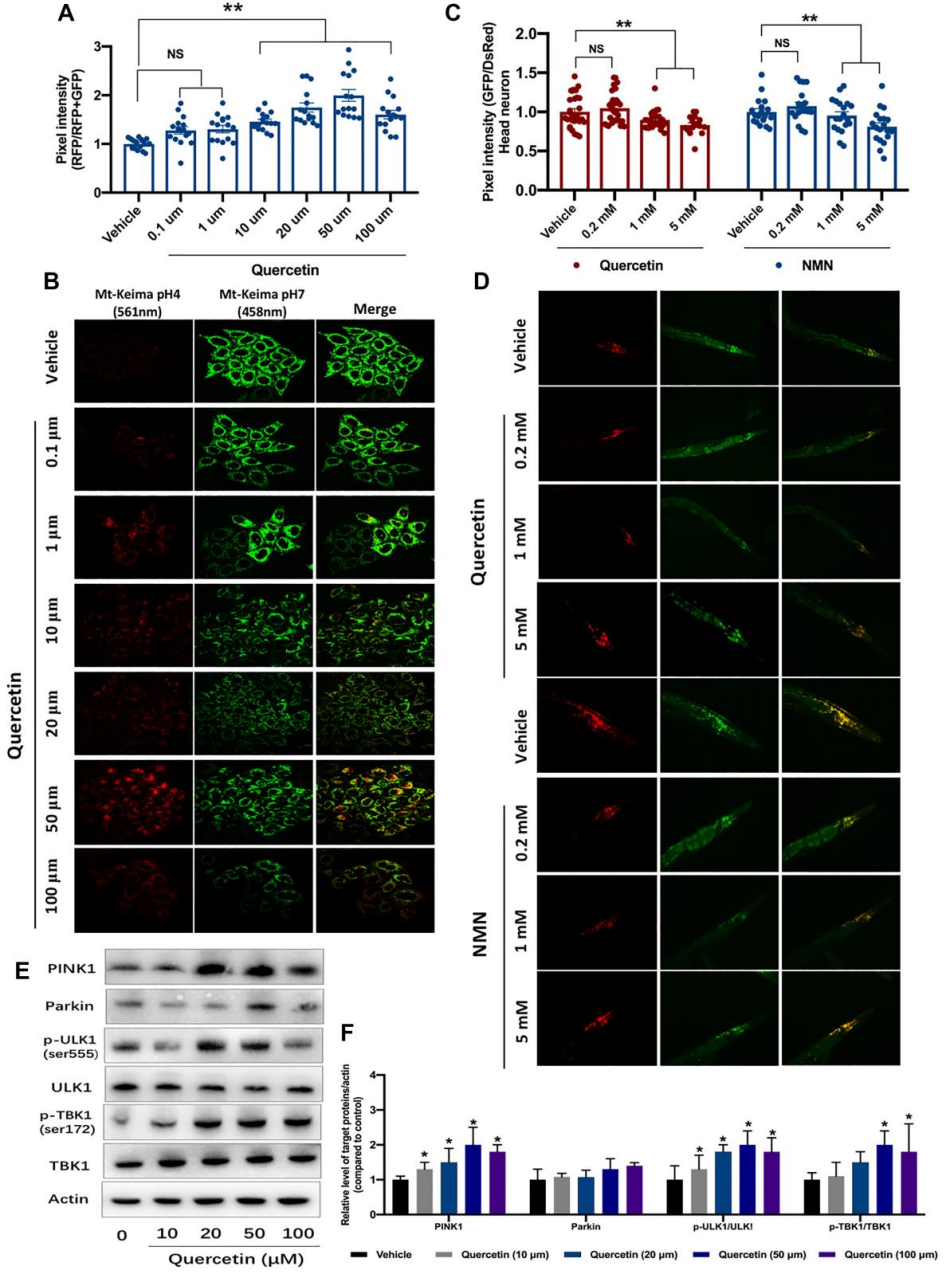
**Quercetin induces the mitophagy ability *in vitro* and *in vivo*.** (**A**) Evaluation of mitophagy in vehicle- and quercetin (0.1 1, 10, 20, 50 and 100 μM)-treated HeLa cells expressing mt-Keima. Ratios indicating relative levels of mitophagy were quantified. (**B**) Representative images of (**A**). (**C**) Transgenic animals expressing the mt-Rosella biosensor in neuronal cells were treated with quercetin and NMN. Relative levels of neuronal mitophagy are expressed as the ratio between pH-sensitive GFP fluorescence intensity and pH-insensitive DsRed fluorescence intensity (*n* = 35 nematodes per group). (**D**) Representative images of (**C**). (**E**, **F**) Changes of designated mitophagy proteins in Hela cells with or without quercetin treatment. Data are expressed as mean ± SEM. ^*^P < 0.05 compared to the control (ANOVA followed by Dunnett's multiple-comparison post hoc tests). For all nematode experiments, two to three independent experiments were performed.

Due to the close interplay between mitochondrial biogenesis and mitophagy, we asked if quercetin increases mitophagy. In this paper, we used mt-Keima mitophagy reporter to verify changes of mitophagy by quercetin. We found quercetin (especially at 10, 20, 50 μM) increased the mt-Keima signal, indicating higher mitophagy (Figure 1A and 1B), and the mt-Keima signal can be abrogated by the *Pink1* siRNA (Supplementary Figure 1). Moreover, we used the transgenic *C. elegans* neuronal mt-Rosella to reflect the mitophagy induction [24]. Quercetin (1 and 5 mM) could reduce the GFP/DsRed value of Rosella fluorescence, indicating stimulation of mitophagy. Meanwhile, the ability of mitophagy induction by quercetin is comparable when comparing with the nicotinamide mononucleotide (NMN), which as a positive control mitophagy inducer (Figure 1C and 1D). In conclusion, Quercetin can induce mitophagy across species. To uncover the potential molecular mechanisms by which quercetin induce mitophagy, we treated HeLa cells with quercetin, at doses ranging from 10 to 100 μM. The results showed that quercetin increased the protein levels of a series of mitophagy-related proteins, including PINK1, Parkin, p-ULK1(Ser555)/ULK1, p-TBK1(Ser172)/TBK1 and LC3B (Figure 1E, 1F and Supplementary Figure 2).

### Effects of quercetin on neurotoxic-induced injury and mitochondria parameters in PC12 cells

**Figure 2:**
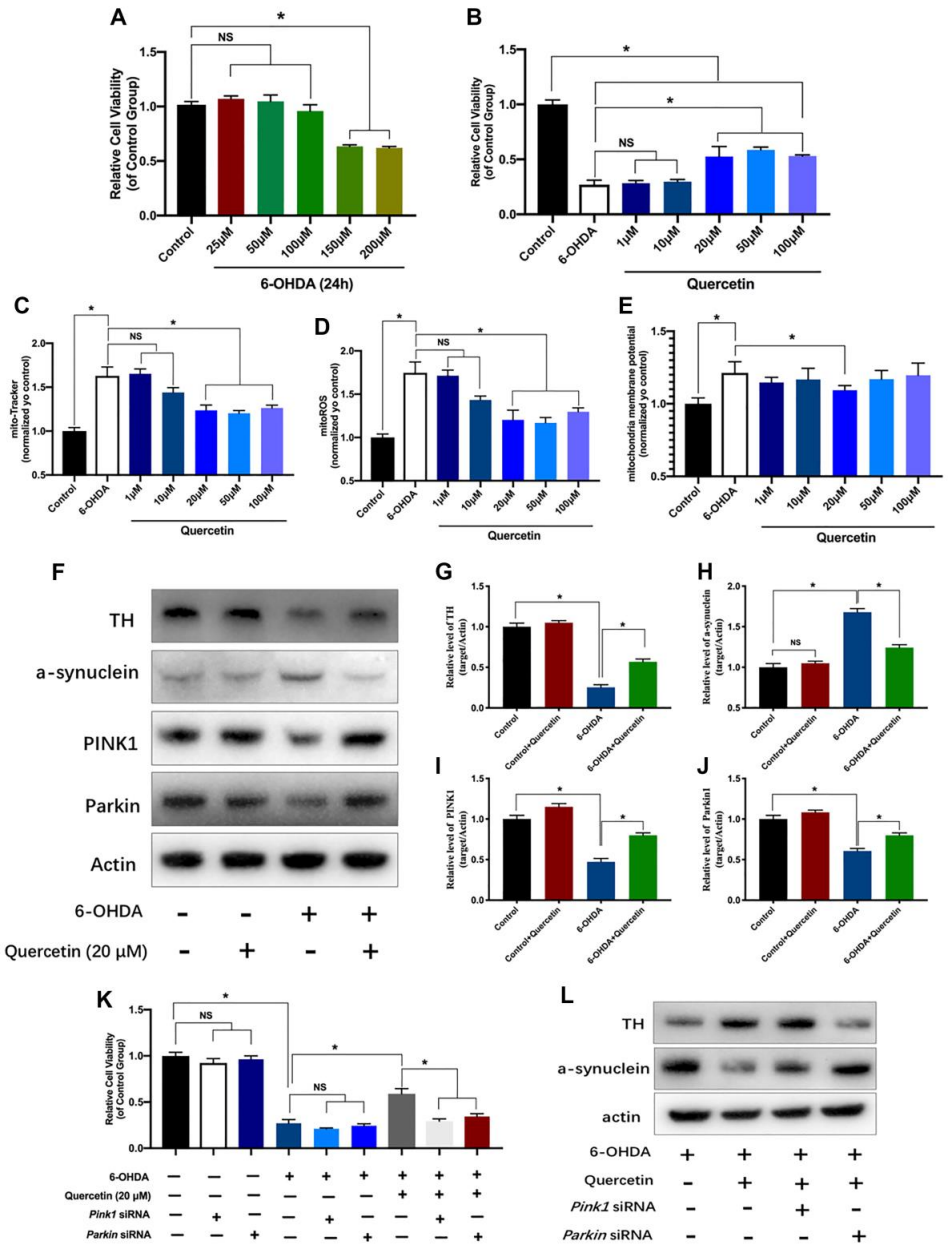
**Neuroprotective effects of quercetin on neurotoxic-induced injury and mitochondria parameters in PC12 cells via PINK1 and Parkin mitophagy pathway.** (**A**) Effects of different concentration of 6-OHDA on PC12 cells viability. (**B**) After pretreated with different concentration of quercetin (1, 10, 20, 50, 100 μM), the cells were incubated with 6-OHDA (150 μM) and different concentration of quercetin for 24 h. Cell viability assessed using the CCK8 assay. Data are expressed as mean ± SEM. *n* = 5–6 wells for each group. ^*^P < 0.05 compared to the control. Evaluation of mitochondria function parameters, such as mitochondria contents (**C**), mitochondria ROS (**D**), and mitochondria membrane potential (**E**). (**F**) Changes of Tyrosine Hydroxylase (TH), a-synuclein and mitophagy proteins (PINK1 and Parkin) in HeLa cells with or without quercetin treatment. (**G**–**J**) Quantification of (**F**). (**K**) Cell viability assessed using the CCK8 assay with or without Pink1 and Parkin siRNA treatment. (**L**) The protein levels of TH and a-synuclein in different groups. Data are expressed as mean ± SEM. ^*^P < 0.05 compared to the designated control (ANOVA followed by Dunnett's multiple-comparison post hoc tests).

After a PD cell model was established with the administration of 6-OHDA (Figure 2A), quercetin was used to treat 6-OHDA incubated PC12 cells. As shown in Figure 2B, the cell viability was increased with the quercetin treatment at 20, 50 and 100 μM (Figure 2B). Meanwhile, we found there was no significant difference between 20, 50 and 100 μM (Figure 2B). Hence, we choose the 20 μM of quercetin for follow experiments. Mitochondrial impairment, cellular energy failure and oxidative stress, may be important mechanisms in PD pathophysiology. Hence, we further tested mitochondrial performance alterations under 6-OHDA injection and quercetin administration. We first conducted MitoTracker fluorescent staining to reflect the mitochondria contents and found an obvious reduction in the 6-OHDA group compared with the control, indicating the fragment damaged mitochondria. The contents and integrity of the mitochondria rescued by Quercetin administration (Figure 2C). In addition, we measured that 6-OHDA caused increased ROS release and MMP in PC12 cells, and these changes were significantly reversed by quercetin in a dose-dependent manner (Figure 2D and 2E).

### The neuroprotection of quercetin *in vitro* dependent on the Pink1-parkin mitophagy pathway

Quercetin alone did not show either increase or decrease the TH levels while 6-OHDA treatment resulted in a dramatic decrease the Tyrosine Hydroxylase (TH) levels, as compared to the control group, and this change was partially reversed by quercetin 20 μM (Figure 2F, 2G). In parallel, a-synuclein increased in the PD cell group, and quercetin could obviously decrease the a-synuclein contents (Figure 2F, 2H). We further evaluated the efficacy of quercetin in mitophagy relevant markers. The activities of PINK1 and Parkin were significantly reduced in response to 6-OHDA. However, quercetin was able to abrogate 6-OHDA-induced low mitophagy activity (Figure 2F, 2I, 2J). Taken together, our data suggest that quercetin has a potent protective effect against 6-OHDA-mediated neuron toxicity and mitochondria dysfunction *in vitro* through the mitophagy induction. To verify this idea, we knockdown the *Pink1* and *Parkin* using siRNA, and found the neuroprotective of the cell viability with the quercetin treatment can be revered by the either *Pink1* or *Parkin* siRNA (Figure 2K). Moreover, the levels of the TH and a-synuclein aggregates as well offset by the Pink1 or parkin siRNA (Figure 2L). Altogether, we concluded the protectives effects of quercetin likely dependent the PINK1-Parkin mitophagy pathway.

### Quercetin improves the neurochemical and parkinsonian disability score in 6-OHDA-lesioned PD rats

**Figure 3:**
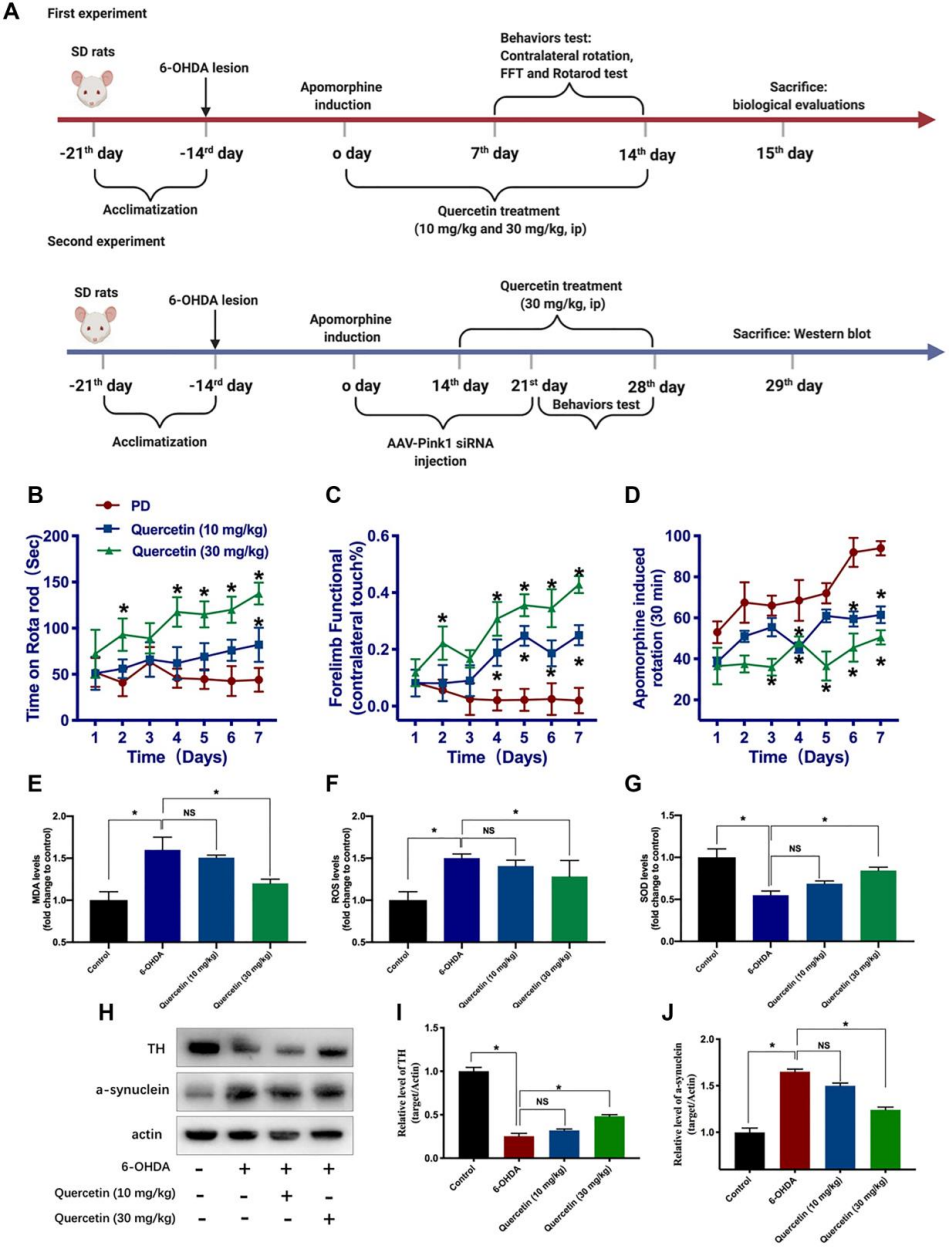
**Quercetin improves the neurochemical and parkinsonian disability score in 6-OHDA-lesioned PD rats.** (**A**) Schema of the two part experiments. Rats were rated for rotarod test (**B**), forelimb function (**C**), and apomorphine induced contralateral rotation (**D**) (^*^P < 0.05 compared to the designated control, *n* = 8 for each group, two-way ANOVA test followed by Bonferroni’s post hoc test). Assessment of the oxidative markers with or without quercetin treatment in substantia nigra, such as MDA (**E**), ROS (**F**), and SOD (**G**) (*n* = 4 for each group). (**H**) The protein levels of TH and a-synuclein in different groups (*n* = 4 for each group). (**I**) Quantification of the TH protein level in each group. (**J**) Quantification of the a-synuclein protein level in each group. Data are expressed as mean ± SEM. ^*^P < 0.05 compared to the designated control (ANOVA followed by Dunnett's multiple-comparison post hoc tests).

Based on the aforementioned results, quercetin has been shown to protect against neuron death in *in vitro* cellular model of PD. To verify its protective effects in 6-OHDA-lesioned PD rats model, as shown in the schema of the experiment (Figure 3A), apomorphine-induced contralateral rotation was to determine the successful establishment of PD rat model and behaviorally examine the neuroprotective effects of quercetin in PD rats. In terms of rotarod test, quercetin (30 mg/kg) treatment could increase the time on the rotarod compared with PD rats (Figure 3B). However, there was no difference between the quercetin (10 mg/kg) and PD group, indicating low dose of quercetin did not influence the rotarod test performance. Parallelly, as depicted in Figure 3C, forelimb function score was obviously ameliorated by quercetin treatment. Quercetin treatment increased the percentage of lesioned forelimbs utilized versus PD rats after the second day. Meanwhile, the improvement in forelimb function was more apparent in the quercetin 30 mg/kg than 10 mg/kg group (Figure 3C). We found that quercetin significantly reduced contralateral rotation in 6-OHDA-lesioned rats (Figure 3D). With regards to the quercetin-L (10 mg/kg) and quercetin-H groups (30 mg/kg), the protective effect of quercetin on contralateral rotation in PD rats is dose-dependent. In brief, based on the behavior results, the 6-OHDA-induced parkinsonian disability scores were significantly reversed by the quercetin daily treatment. In addition, we measured the activity of three important indicators of oxidative stress, MDA, SOD and ROS. 6-OHDA caused increased MDA and ROS release and decreased SOD activity in PC12 cells, and these changes were significantly reversed by quercetin in a dose-dependent manner (Figure 3E–3G). Moreover, western blot analysis showed that 6-OHDA reduced TH in the substantia nigra (SN), but that reduction was attenuated in the 6-OHDA plus quercetin 30 mg/kg group. Similarly, quercetin prevented 6-OHDA-induced augmentation of a-synuclein expression in the midbrain (Figure 3H–3J).

### Pink1-Parkin-mediated mitophagy pathway implicated in the function of quercetin in PD rats

**Figure 4:**
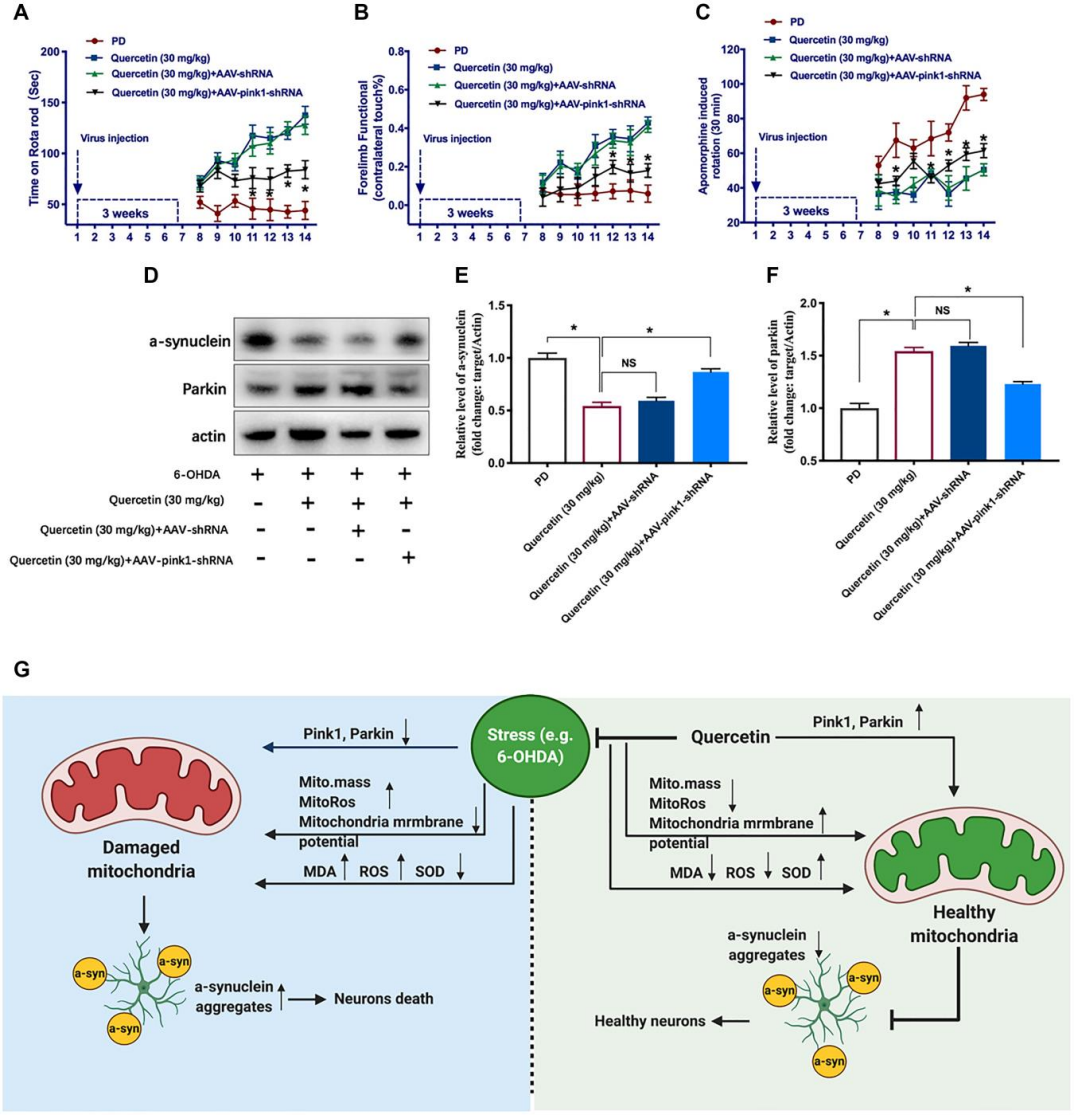
**Pink1-mediated mitophagy pathway implicated in the function of quercetin in PD rats.** In each group, rats were rated for rotarod test (**A**), forelimb function (**B**), and apomorphine induced contralateral rotation (**C**) (^*^P < 0.05 compared to the designated control, *n* = 6 for each group, two-way ANOVA test followed by Bonferroni’s post hoc test). (**D**) The protein levels of a-synuclein and Parkin in different groups (*n* = 6 for each group). (**E**) Quantification of the a-synuclein protein level in each group. (**F**) Quantification of the Parkin protein level in each group. (**G**) The working model and potential mechanism of quercetin in the 6-OHDA-lesioned PD models. Data are expressed as mean ± SEM. ^*^P < 0.05 compared to the designated control (ANOVA followed by Dunnett's multiple-comparison post hoc tests).

To explore PINK1-Parkin mitophagy pathway as a potential mechanism contributing to the quercetin-induced neuroprotection in the PD models, we performed AAV-mediated knockdown *pink1* gene in the striatum and test the behavior phenotypes. We generated AAV vectors encoding for either a short hairpin RNA (shRNA) to block PINK1 expression (*Pink1^−^*^/*−*^, AAV-*Pink1*-shRNA) or a shRNA against firefly luciferase (AAV-shRNA) as a negative control. In this experiment set, we found quercetin (30 mg/kg) treatment could increase the time on the rotarod compared with PD rats, and genetic deletion of PINK1 (AAV-Pink1-shRNA) significantly offset the protect effects of quercetin treatment (Figure 4A). However, there was no difference between the quercetin and quercetin plus AAV-shRNA group. In addition, as shown before, quercetin treatment increased the percentage of lesioned forelimbs utilized versus PD rats, and the phenotype also reversed by the AAV-*Pink1*-shRNA, rather than in the AAV-shRNA group (Figure 4B). Analogously, the protective effect of quercetin on contralateral rotation in PD rats is dependent on the PINK1 expression (Figure 4C). Taken together, these results showed that quercetin improved the PD rats performances maybe via PINK1-Parkin mitophagy pathway. To elucidate the mitophagy induction inhibit a-synuclein aggregates by the 6-OHDA, we examined the western blot to test the a-synuclein levels. Interestingly, quercetin arrested 6-OHDA-induced augmentation of a-synuclein expression in the midbrain is counteracted by the AAV-*Pink1*-shRNA, as well as obviously reduced the Parkin protein expression (Figure 4D–4F). We also summarized the working model of quercetin in the 6-OHDA-lesioned PD models in the Figure 4G.

## DISCUSSION

Mounting evidence suggests that mitochondrial dysfunction and impaired mitophagy contribute to PD and other aging-related diseases [5, 9, 29]. Our results provide evidence that mitophagy defects have a critical role in PD development and progression. Here, we report PC12 cells under 6-OHDA treatment exhibit mitochondrial dysfunction characterized by decreased mitochondrial content, increased oxidative stress and reduced mitochondrial membrane potential, all of which are improved by quercetin treatment. As mentioned before, quercetin has profound effects on PINK1-Parkin expression in 6-OHDA-treated PC12 cells and rats, prevents 6-OHDA-induced neuronal loss and behavioral deficits, and induces mitophagy in mt-Keima-overexpressing HeLa cells and mito-Rosella-expressing *C. elegans*. Moreover, the neuroprotective effect of quercetin in an *in vitro* cellular PD model is reduced by either *Pink1* or *Parkin* siRNA, and the function of quercetin in PD rats *in vivo* is also reversed by *Pink1* shRNA. Therefore, it is reasonable to assume that the increase in mitophagy, at least in part, contributes to the neuroprotective effects of quercetin in the 6-OHDA-induced PD models. Moreover, our findings demonstrate that administration of quercetin relieves 6-OHDA-induced progressive PD-like motor behaviors, mitigates neuronal death, and reduces mitochondrial damage and oxidative markers. In summary, this paper provides proof-of-concept evidence that quercetin exerts a partial protective effect against the deleterious effects of 30 mg/kg 6-OHDA. In addition, the findings extend the “mitochondrial cascade hypothesis” of PD by linking α -synuclein deposition to defective mitophagy. Induction of mitophagy through PINK1-Parkin via quercetin supplementation reduces the number of a-synuclein aggregates. In summary, our study is the first to show that quercetin prevents neurotoxicity by reducing 6-OHDA-induced activation of PINK1-Parkin-dependent mitophagy. These results further support previous demonstrations of mitochondrial dysfunction or impaired mitophagy implicated in PD pathogenesis.

Some parkinsonian toxins, such as MPP+ and 6-OHDA, like pesticide paraquat, also can stimulate mitophagy via the PINK1–parkin pathway. 6-OHDA activates the process by promoting the externalization of cardiolipin, which recruits the autophagic machinery via a direct interaction with LC3. At the molecular level, exploring the function of genes mutated in hereditary PD yields insight into disease etiology and reveals new pathways in cell biology. Among them, PINK1 and Parkin, normally work together in the close pathway to govern mitochondrial quality control [30]. When mitochondria under stresses, PINK1 accumulates on the outer membrane of mitochondria (OMM), then activates Parkin's E3 ubiquitin ligase activity and follow recruits Parkin to the OMM [31]. Subsequent, Parkin ubiquitinates OMM proteins to trigger selective autophagy, refer to mitophagy. However, the intricately point is that, in mice, both *PINK1^−/−^* and *Parkin^−/−^* show no substantial PD-relevant behavior phenotypes [32]. More than that, *in vivo* evidence reported basal mammalian mitophagy occurred independently of PINK1 [33], indicating yet-to-be-discovered pathways orchestrating mammalian mitochondrial integrity in a context-dependent fashion, or the paradoxical phenomenon maybe due to compensation of the loss of PINK1-dependent mitophagy by other pathways under physiological conditions [7]. Notably, a recent study reported that under acute or chronic mitochondrial stress conditions, there was a strong inflammatory phenotype in both *PINK1^−/−^* and *Parkin^−/−^* mice, indicating a role for PINK1-Parkin-mediated mitophagy in restraining innate immunity [34]. Altogether, these data suggest that the PINK1-Parkin-dependent mitophagy may be unessential at physiological conditions but is dispensable at stress or pathological conditions [5]. Based on our present study, we found the level of mitophagy marker PINK1 and Parkin were lower in PD group compared with sham group *in vivo* and *in vitro*, and quercetin could obviously reverse such phenomenon. Both from our results and previous studies displayed that mitophagy dysfunction is a hallmark pathology implicated in PD pathogenesis.

As a natural phytochemical, quercetin, one of the most abundant polyphenolic flavonoids, is present in fruits and vegetables and displays many health-promoting biological effects in a wide range of diseases, such as cancer, cardiovascular disease, cataract, inflammation, diabetes, and nervous system disorders [31]. Quercetin acts as a direct antioxidant that neutralizes oxidative stress by scavenging ROS; when quercetin reacts with free radicals, it converts them to a more stable condition with less reactivity, thus preserving cell viability [31]. In addition, quercetin plays a vital role in reducing the levels of proinflammatory cytokines such as TNF-α, IL-1β, IL-6, and IL-8 and suppresses the release of NF-κB nuclear factor, thereby preventing its entry into the nucleus; all of these functions are involved in the anti-inflammatory properties of quercetin [31]. In this paper, we found that quercetin improved mitochondrial quality control and protected neurons in 6-OHDA-induced PD models by activating the PINK1-Parkin mitophagy pathway. Notably, a major barrier to the clinical efficacy of quercetin is its poor bioavailability. However, several studies have shown that quercetin can still pass through the blood brain barrier (BBB) due to its lipophilic properties and act as a neuroprotectant [35], even though the concentration and distribution of quercetin in the brain are lower than those in other tissues [31]. Moreover, the low bioavailability of quercetin has led researchers to attempt various quercetin-loaded nanoparticles to overcome these limitations [36]. One limitation of this paper was that we used pure quercetin. In the future, we should attempt to use advanced drug delivery methods to enhance the bioavailability of quercetin in the brain.

In summary, the neuroprotective effects of quercetin in the 6-OHDA-lesioned PD models have for the first time been associated with direct effects of quercetin on PINK1-Parkin mitophagy pathway that lead to eliminate a-synuclein aggregates and relieve behavior phenotypes. These findings expand our knowledge about the protective mechanisms of quercetin in 6-OHDA PD models and provide additional targets for therapeutic interventions in PD.

## Supplementary Material

Supplementary Figures
