# Supplementary material for: Administration of quercetin improves mitochondria quality control and protects the neurons in 6-OHDA-lesioned Parkinson's disease models
Source: Aging (Albany NY). 2021 Apr 20;13(8):11738–51. doi: 10.18632/aging.202868 (PMC8109056; doi:10.18632/aging.202868)
Supplement: Supplementary Figures [file aging-13-202868-s001.pdf]

SUPPLEMENTARY FIGURES

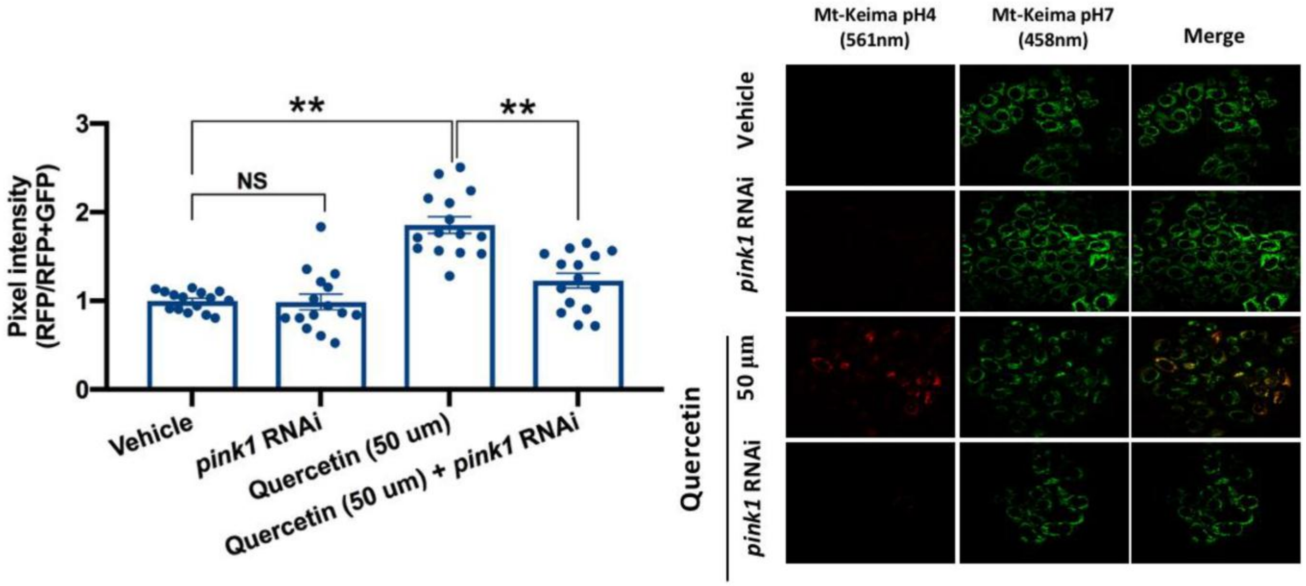

Supplementary Figure 1. Evaluation of mitophagy in vehicle- and quercetin (50 μM)-treated HeLa cells expressing mt-Keima with *pink1* siRNA. Ratios indicating relative levels of mitophagy were quantified. Data are expressed as mean ± SEM. $^{**}P < 0.01$.

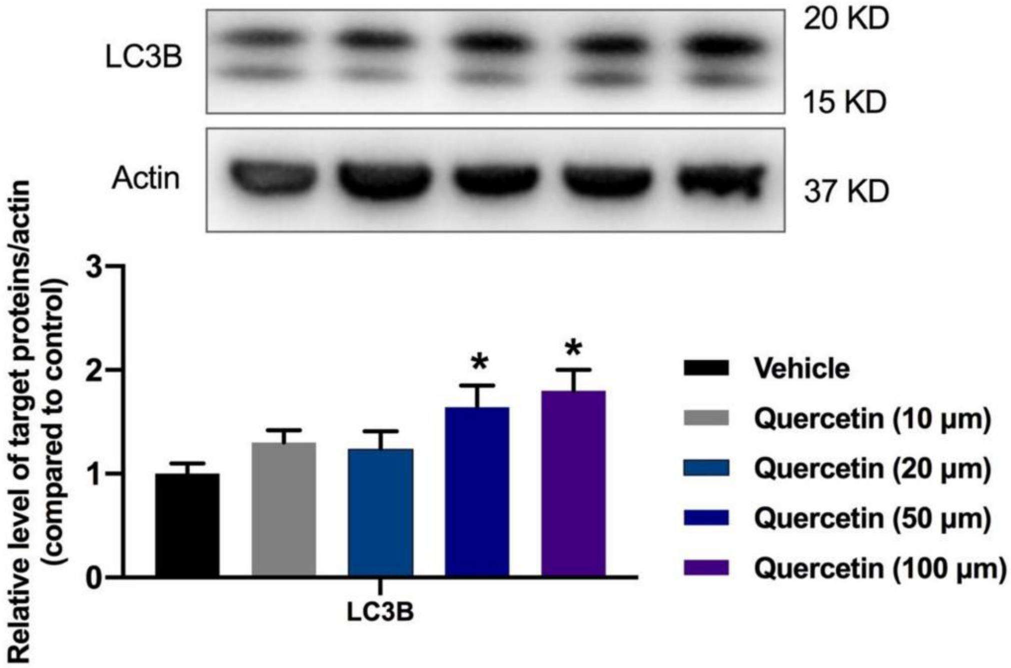

Supplementary Figure 2. Changes of designated mitophagy proteins in Hela cells with or without quercetin treatment. Data are expressed as mean ± SEM. $^{*}P < 0.05$ compared to the control.
